# The Mediating Role of Self-Efficacy and Outcome Expectations in the Relationship Between Peer Context and Academic Engagement: A Social Cognitive Theory Perspective

**DOI:** 10.3390/bs15050681

**Published:** 2025-05-16

**Authors:** Getachew Tassew Woreta, Girum Tareke Zewude, Krisztián Józsa

**Affiliations:** 1 Department of Psychology, Wollo University, Dessie 1145, Ethiopia; girum.tareke@wu.edu.et; 2 Institute of Education, University of Szeged, 6722 Szeged, Hungary; 3 Institute of Education, Hungarian University of Agriculture and Life Sciences, 7400 Kaposvár, Hungary

**Keywords:** peer context, academic engagement, self-efficacy, outcome expectations, peer influence, high school, Ethiopia, social cognitive theory

## Abstract

Student engagement in learning has well-recognized positive effects on both academic and non-academic aspects of development. However, there has been limited research on the factors that shape it. This study examined the influence of peers’ academic norms, educational aspirations, and effort socialization on students’ academic engagement, placing self-efficacy and outcome expectations as mediators. Grounded in Bandura’s social cognitive theory, data were collected cross-sectionally from 596 high school students (male = 315) in Ethiopia. The results of the path analysis demonstrated a good model-data fit. Peers’ academic norms, educational aspirations, and effort socialization positively predicted academic engagement. Bootstrap analysis with 5000 samples revealed that academic self-efficacy (β = 0.022, BC 95% CI = [0.008, 0.041], *p* < 0.01) and outcome expectations (β = 0.053, BC 95% CI = [0.028, 0.086], *p* < 0.001) partially mediated the relationship between peer educational aspirations and students’ academic engagement. The partial mediated effects of peers’ academic norms on academic engagement via self-efficacy (β = 0.030, BC 95% CI = [0.014, 0.054], *p* < 0.001) and outcome expectations (β = 0.037, BC 95% CI = [0.014, 0.062], *p* < 0.01) were also significant. Additionally, peer effort socialization showed significant positive indirect effects on academic engagement, mediated by academic self-efficacy (β = 0.024, BC 95% CI = [0.009, 0.044], *p* < 0.01) and outcome expectations (β = 0.078, BC 95% CI = [0.050, 0.112], *p* < 0.001). Overall, the mediation analysis revealed that outcome expectations and self-efficacy partially mediated the link between academic engagement and the peer context, highlighting the importance of these mediators in enhancing student engagement.

## 1. Introduction

Despite variation in conceptualizations and measurements, studies have demonstrated that student engagement is linked to better academic achievement (e.g., Dogan, 2017; Fung et al., 2018; Hao et al., 2018; Li & Lerner, 2011; Reeve & Lee, 2014; M.-T. Wang & Holcombe, 2010), school completion, a low risk of school dropout and delinquency (Archambault et al., 2009; Fall & Roberts, 2012; Henry et al., 2012; M.-T. Wang & Fredricks, 2014; M.-T. Wang & Peck, 2013), greater persistence in learning (Fredricks et al., 2004), valuable coping skills (Reschly et al., 2008), lower depression (Li & Lerner, 2011), and better well-being (Boulton et al., 2019; Reschly et al., 2008; Salmela-Aro et al., 2009; M.-T. Wang et al., 2015).

Consequently, exploring the potential influences on student engagement should be a key priority for educators and researchers aiming to improve academic outcomes and promote youths’ psychosocial well-being (Alrashidi et al., 2016). However, the attention given to these matters is not as substantial as it deserves, particularly in the Ethiopian context. The absence of empirical data from the Ethiopian school population highlights a significant knowledge gap regarding the representativeness of the student engagement scholarship. Therefore, a study involving the Ethiopian population is imperative to promote inclusivity in research and generate knowledge that is more representative of diverse cultures. Accordingly, this study focused on examining how the peer academic context influences student academic engagement, incorporating self-efficacy and outcome expectations as mediators to address the gaps observed in both global and local contexts.

In Ethiopia, students walk long distances in groups between home and school and regularly gather at home or nearby to collaborate on schoolwork and prepare for tests together (Woreta, 2024). As a result, particularly high school students have more opportunities to frequently contact and spend time with their friends, exchange information about their academic matters, and influence each other’s behavior. Moreover, because of these opportunities, compared to the individualistic Western world, the influence of peer groups in a collective society like Ethiopia on students’ academic motivation and behavior is likely higher. However, the influence of peers on students’ academic-related beliefs, attitudes, motivation, and behavior has received limited attention.

Beyond cultural collectivism, Ethiopia’s political landscape has significantly shaped the educational experience. Shifting government regimes—each with distinct ideological orientations—have resulted in frequent changes in the educational policy, often emphasizing expansion over quality (Kumar et al., 2023). These systematic shifts have created inconsistencies in educational delivery, uneven resource allocation, and regional differences, all of which shape the schooling environment students encounter daily (Carvalho et al., 2022). In such a politically influenced context, peer relations often emerge as stable and accessible sources of academic and emotional support, playing a vital role in students’ learning and motivation.

Academic engagement, in this study, refers to “students’ psychological state of activity that affords them to feel activated, exert effort, and be absorbed during learning activities” (Wong & Liem, 2022, p. 120), including affective, behavioral, and cognitive dimensions. Emotional engagement refers to the degree to which students feel activated and exhibit positive feelings such as interest, enjoyment, vigor, and alertness while they are learning in the classroom or completing school tasks. Behavioral engagement refers to the extent to which students exert effort and remain persistent in their learning activities, with effort and persistence as its key features. Cognitive engagement, on the other hand, refers to the degree to which students are absorbed in the course of learning activities, marked by a high level of attentiveness and reduced awareness of distractions (Wong & Liem, 2022). Ben-Eliyahu et al. (2018) assert that cognitive engagement actually represents thinking and paying attention. Even if engagement is a multidimensional construct, its total score was used for the analysis in this study.

## 2. Theoretical Framework

In an educational context, social cognitive theory (SCT: Bandura, 1986, 1997) provides useful conceptual foundations for understanding how students’ academic-related motivations and behaviors are influenced by the environment and personal factors. A key to SCT is the premise that the interactions among the environment, individuals, and behavior are pivotal in shaping human functioning and that the social context determines the beliefs and behaviors that people form and develop (Bandura, 1997; Egele & Stark, 2024). The roles of personal factors, specifically, self-efficacy and outcome expectations, in mediating the influence of the environment on human behavior are also stressed in SCT.

Consistent with SCT, in the present study, students’ learning engagement (i.e., behavior) is viewed as influenced by personal (i.e., self-efficacy and outcome expectations) and environmental (i.e., peer contexts) factors. Self-efficacy, which is considerably underscored as an important determinant of human behavior, refers to “people’s judgments of their capabilities to organize and execute courses of action required to attain designated types of performances” (Bandura, 1986, p. 391). This means that students’ beliefs in their capabilities play a significant role in determining their behavior, persistence against obstacles, effort exerted, thought patterns, and emotional reactions in connection with pursuing a certain level of education, as posited in SCT (Schunk & DiBenedetto, 2022). The other influential construct within the theory is outcome expectations, which represent the anticipated results or desired consequences of deliberate actions taken by individuals, with the assumption that individuals tend to participate in actions they believe will yield favorable and desirable results while refraining from actions where they do not expect positive outcomes (Bandura, 2001; Schunk & DiBenedetto, 2022). However, the construct of outcome expectations has received negligible focus in the academic motivation, engagement, and achievement literature. It seems essential to understand that self-efficacy does not matter if the individual does not have positive outcome expectations for courses of action to be carried out. If a student does not anticipate positive outcomes from pursuing education, the student’s decision is not to pursue his/her education, despite having a high level of academic self-efficacy. Therefore, as highlighted by Bandura (1986, 1997), self-efficacy and outcome expectations determine human behavior, such as academic engagement (Ghbari et al., 2024). People form self-efficacy and outcome expectations associated with particular actions by observing environmental situations and their own actions’ results (Bandura, 1986). Within a peer context, through symbolic thinking, students envision potential consequences and adjust their behavior. While the focus of research has been the sources of efficacy beliefs, outcome expectations are also influenced by similar sources (Fouad & Guillen, 2006; Lent et al., 1994; Woreta, 2024).

**Figure 1:**
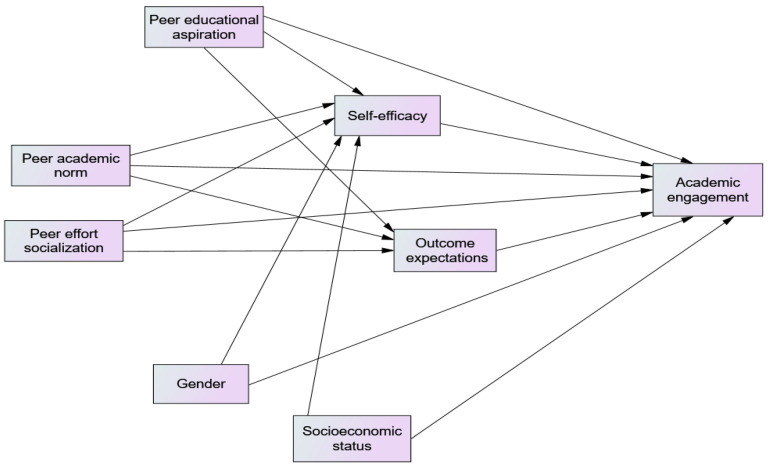
A proposed mediation model and peer matter engagement model.

Therefore, founded on SCT, the peer context is assumed to influence student learning engagement both directly and indirectly through outcome expectations and self-efficacy. The peer context could encompass multiple aspects. However, the present study considered peers’ academic norms, educational aspirations, and effort socialization as the most influential components of peer contexts in shaping the self-efficacy, outcome expectations, and academic engagement of adolescent high school students. Founded on SCT and prior empirical findings, the proposed model (Figure 1) hypothesis is that students are more likely to be engaged in their learning when they (a) experience supportive and encouraging peer contexts, (b) believe that they can accomplish learning-related tasks (self-efficacy), and (c) believe that accomplishing these tasks will lead to positive outcomes (outcome expectations). Although SCT posits that the environment, individuals, and behavior all interact and influence each other, this study focused on the theory’s general prediction that the peer context influences students’ learning engagement both directly and indirectly through self-efficacy and outcome expectations, not on the specific reciprocal relationships among these variables.

These dynamics are particularly salient in the Ethiopian context, where political conditions have historically influenced educational structures, classroom conditions, and resource distribution (Adamu, 2015). From the perspective of social cognitive theory, these macro-level political factors constitute part of the broader environmental context that shapes students’ beliefs and behaviors (Herut & Dube, 2022). Specifically, in politically driven systems with under-resourced schools and limited teacher support, students may rely more heavily on peer interactions for self-efficacy beliefs and outcome expectations, making the peer context a critical mediator of engagement outcomes.

### 2.1. Peer Context and Academic Engagement

Peers either enhance or discourage the academic attitudes, values, and behaviors of individuals who belong to them (Shin & Ryan, 2014; M.-T. Wang et al., 2018) and these in turn collectively determine to what degree each student engages in academic tasks. Over time, peers socialize with each other and change their academic performance, behaviors, enthusiasm, and engagement (Altermatt & Pomerantz, 2003; Kindermann, 2007; Ryan, 2001; Shin, 2022) because modeling or the exchange of persuasive messages affects one’s thought, actions, or feelings (Kindermann & Gest, 2009; Ryan et al., 2019). Friends are similar in numerous characteristics, such as academic efforts (Ryan, 2001; Shin & Ryan, 2014), disruptive behavior (Shin & Ryan, 2014), and values and interests (Ryan, 2001; Shin & Ryan, 2014), which suggest the degree to which peers matter for socializing students’ academic engagement and the opportunities peers have to model and influence each other’s academic behaviors and motivation (Palacios & Berger, 2022; Wentzel, 2009). However, Steenberghs et al. (2021) note that researchers have not shown adequate attention to how peer contexts influence students’ academic engagement. Recently, Woreta (2024) examined the role of peer academic socialization—conceptualized to include academic norms, educational aspirations, and effort socialization—in shaping academic engagement. However, the study did not specifically assess the independent predictive power of these aspects on student academic engagement, highlighting a gap that warrants further investigation.

The positive academic behaviors prevalent in a particular peer group, such as attending class, completing homework, and aiming and valuing higher academic results, denote peer academic norms (McCormick & Cappella, 2014). According to Steenberghs et al. (2021), students’ positive academic behaviors, such as academic engagement, increase if friends and classmates have more compliant academic behaviors. This highlights that the academic norms prevailing in the peer group are imperative to enhance student academic engagement. Peers’ effort socialization refers to a process that involves messages emphasizing the benefits of sustained efforts and hard work in academic settings, as well as linking a poor academic performance with insufficient effort among individuals within the same age group or social circle (Woreta, 2024). As a socialization process inspires the need to exert maximum academic effort for greater success, peers’ messages of effort are important in shaping individuals’ motivation and mindset toward effort and success. Therefore, it is posited that peer contexts that emphasize and model the value of effort and hard work play crucial roles in facilitating students’ academic engagement.

During their high school years, students start to reflect on their academic and career pathways (Eccles et al., 2004; Kiuru et al., 2007; Vernon & Drane, 2020) because they usually discuss their education-related views and aspirations with each other. This highlights the importance of others’ aspirations in the peer group to socialize students’ education-related beliefs and behaviors, such as academic engagement, particularly in Ethiopian culture, which is known for being collectivistic. Accordingly, including peers’ aspirations as one key aspect of the peer context and understanding its influence on student academic engagement is found to be important. This study, therefore, examined the unique effects of each aspect of the peer context (i.e., peers’ educational aspirations, effort socialization, and academic norms) for cultivating high school students’ learning engagement. That is, each dimension’s independent influence was in focus, rather than the combined influence of the peer academic context as a latent construct.

### 2.2. Self-Efficacy, Outcome Expectations, and Academic Engagement

Research at different educational settings indicates that students with high academic self-efficacy beliefs exhibit greater academic engagement (Alemayehu & Chen, 2023; Guo et al., 2025; Huang & Wang, 2023; Lin, 2021; Q. Liu et al., 2023; R. D. Liu et al., 2018; Martin & Rimm-Kaufman, 2015; Meng & Zhang, 2023; Navarro et al., 2019; Rimm-Kaufman et al., 2015; Shao et al., 2025; Wilson et al., 2015). A recent study conducted in Ethiopia also supports this finding (Woreta, 2024). However, as highlighted in SCT, self-efficacy alone is not sufficient to motivate human behaviors; outcome expectations also have a crucial role in determining one’s actions. Supporting this view, M.-T. Wang and Degol (2014) argue that students with strong ability beliefs may not put in any effort if they believe that learning will not be rewarded in their current circumstances. Similarly, Fouad and Guillen (2006) and Dawkins et al. (2021) suggest that outcome expectations uniquely contribute to an individual’s engagement in a particular behavior, beyond self-efficacy beliefs. High academic self-efficacy combined with positive outcome expectations leads to greater satisfaction and intentions to remain enrolled in studies, even under high academic and life demands (Bergin & Jimmieson, 2017; Hechenleitner-Carvallo et al., 2019). Studies in Ethiopia and Malaysia found that self-efficacy and outcome expectations significantly and positively mediated the relationship between the peer context and academic engagement (Benlahcene et al., 2024; Woreta, 2024). A study in China also showed that peer relationships were directly and indirectly associated with learning engagement throughout the mediating roles of self-efficacy and academic resilience (Shao & Kang, 2022).

Although empirical evidence on the link between outcome expectations and student engagement in learning activities is limited, studies have found that outcome expectations positively contribute to academic engagement in high school students (Woreta, 2024) and undergraduate engineering students (Navarro et al., 2019). Based on SCT’s conceptualization and propositions, the role of outcome expectations in influencing career-related outcomes, such as interests, goals, persistence, and performance (e.g., Byars-Winston et al., 2010; Kozlowski & Fouad, 2023; Lent et al., 1994, 2016, 2013) has been well researched, but it has received limited attention in student engagement studies.

### 2.3. Peer Context, Self-Efficacy, and Outcome Expectations

A peer group is an educationally relevant socialization context where students experience other-focused sources of academic self-efficacy, such as vicarious experiences and verbal and social persuasions (Bandura, 1997; Schunk & Pajares, 2009; Vollet & Kindermann, 2020). Particularly during adolescence, the peer context exerts increasing influence (Kindermann, 2007) and becomes more important in shaping educationally relevant factors such as confidence in one’s academic ability (Lan, 2023). Peer groups provide a context in which students can enhance their academic self-efficacy (Gebauer et al., 2020). In a peer context, observing others’ positive actions and behavior enhances self-efficacy (Schunk & Pajares, 2009). Peer verbal encouragement and social persuasion also enhance students’ belief in their ability to perform a particular task or course of action (Bandura, 1997; Gebauer et al., 2020).

Bandura (1986, 1997) posits that individuals form outcome expectations associated with particular actions by observing environmental circumstances and events, as well as the actual results of their actions. Lent et al. (1994) and Gerbino (2020) specifically proposed that outcome expectations stem from sources akin to those shaping self-efficacy beliefs. A meta-analysis study found that vicarious learning and verbal persuasion were significant predictors of outcome expectations (Sheu et al., 2018), backing up Lent et al.’s (1994) idea.

Cultures may determine how individuals understand and evaluate socially conveyed sources of self-efficacy and outcome expectations differently (M.-J. Liu et al., 2022). In a cross-cultural study, other-oriented sources (vicarious experience and social persuasion) significantly predicted the Indian–Canadian students’ self-efficacy, but they did not significantly predict Anglo–Canadian students’ self-efficacy (Klassen, 2004). Ahn et al. (2016) also reported that verbal and social persuasion were the strongest factors in shaping efficacy beliefs among students from collectivistic cultures. So, for the current participants, it was hypothesized that peers’ academic norms, aspirations for educational attainment, and effort socialization would influence academic engagement, partially mediated by self-efficacy and outcome expectations.

### 2.4. The Current Study

While the literature documents the positive influence of academic engagement on student development across domains, studies examining the factors that influence it remain limited. Hence, this study aimed to examine the influence of peer context variables, namely, academic norms, educational aspirations, and effort socialization, on students’ academic engagement. Based on SCT’s perspective, the mediating roles of self-efficacy and outcome expectations were in focus to understand how peers matter for students’ academic engagement. The peer context was the focus because it is the most important social context for adolescents’ development in different domains (e.g., Li et al., 2011; Ryan, 2001; Wentzel, 1998). Even positive parenting efforts can be either enhanced or undermined by the characteristics of peer groups. However, a gap remains in understanding how different aspects of the peer context influence student academic engagement. Given that adolescent students spend almost their daily hours in a school environment, surrounded by and interacting with peers, it is arguable that the peer context deserves more attention than other social contexts. Moreover, in collectivistic cultures, the perspectives and actions of others are more influential in shaping human behaviors (Markus & Kitayama, 1991); particularly during adolescence, it can be highly powerful. There is evidence that, in collectivistic cultures, motivational beliefs, like self-efficacy, are fundamentally determined by socialization experiences, such as vicarious experiences and social persuasions from significant others, compared to individualistic cultures (Ahn et al., 2016; Klassen, 2004). Accordingly, for Ethiopian students, examining the relations of peer-focused factors with self-efficacy, outcome expectations, and academic engagement seems to be justifiable.

The academic norms, attitudes, beliefs, and motivation prevailing within a given peer group might be influential in shaping student engagement and performance. A peer context with high-quality caring and support of relatedness might not have positive effects on academic-related beliefs, expectations, and behaviors if the academic characteristics of peers do not comply with the demands of schooling. This study uncovers previously overlooked mechanisms through which the peer influence operates by focusing on the specific aspects of the peer context and addressing structural relations that were not examined in earlier studies.

As mentioned earlier, Woreta (2024) studied the role of academic socialization (by parents and peers) in shaping students’ academic engagement; however, the question of which facet of the peer context distinctively contributes remains open. This gap dictates further study to make clear the component’s unique contributions and to inform relevant interventions that enhance student engagement. A study that examines the distinct influence of each component, rather than the overarching influence of the peer context, can identify the most significant dimensions and facilitate informed, targeted interventions for effective support in educational environments. Focusing just on the underlying factor may obscure the unique contributions of specific dimensions, thus resulting in erroneous results. Nevertheless, examining each dimension individually allows for the discernment of their genuine and independent effects. Furthermore, the effects of each dimension may operate through distinct pathways. Also, as the present study sampled participants from grades 9 to 12, its scientific validity might be considerably enhanced by capturing the entire high school experience and improving the findings’ generalizability beyond the early years. When a study includes the experiences of participants of all high school grades, findings are not limited to a specific subgroup, such as students in earlier or later phases of high school; rather, they become more applicable to a diverse student population. Moreover, in this study, gender and the socioeconomic status were used as covariates to control for unwanted variability that might affect the true relationships between the predictors and outcome variables.

This study also differs from prior studies by examining the applicability of Western theoretical perspectives, often overlooked in non-Western contexts, particularly in Ethiopia. The findings could potentially enhance our knowledge of factors that shape students’ engagement in their learning across society’s cultures and development levels.

Therefore, based on SCT and previous research, it was hypothesized that students who feel their peers or friends have encouraging academic norms, high educational aspirations, and value-sustained efforts and academic success would have higher self-efficacy, form positive outcome expectations for educational pursuits, and become highly engaged in schoolwork. The structural relations of the study variables (i.e., academic norms, educational aspirations, effort socialization, self-efficacy, outcome expectations, and engagement) are depicted in Figure 1. As a whole, this study is presumed to (a) offer empirical evidence for SCT’s cross-cultural applicability, (b) enrich the existing engagement scholarship by offering empirical findings from a population often overlooked in the literature, and (c) put forward interventions that enhance student engagement, which in turn improves their academic performance, particularly in the Ethiopian context where students’ learning engagement and academic performance have been declining significantly to a great extent for a decade.

## 3. Materials and Methods

### 3.1. Participants

This study included 596 high school students from 9th to 12th grade (52.85% male; 93% ethnically Amhara), randomly selected from four public schools in the Dessie and Kombolcha city administrations, Amhara region, Ethiopia. Depending on the student population of each school, the participants’ number ranges from 135 to 163. After data collection from 615 students, the screening process excluded 19 cases either due to incompleteness or significant missing data. As a result, the final sample consisted of 596 students (mean age = 17.4 years, SD = 1.21, range from 15 to 20 years), all of whom were enrolled in regular education programs in public high schools.

For context, Ethiopia’s total secondary school student population (grades 9–12) exceeds three million nationwide, with substantial ethnic and regional diversity (Getacher et al., 2023). While the current sample is representative of students within the Dessie and Kombolcha urban centers in the Amhara region, it may not fully capture the demographic diversity present across other regions and ethnic groups in Ethiopia.

### 3.2. Procedures

First, the research ethics protocol was approved by the Ethics Committee of the Institute of Teachers’ Education and Behavioral Science. Then, we contacted the directors of the respective schools to obtain their consent to contact teachers and students and collect data. Under the researcher’s close supervision, schoolteachers administered the questionnaire in regular classrooms without time limits; informed consent was obtained through signed consent forms from either the students or their parents, depending on the student’s age. Participants were informed not to write any personal identifying variables, such as names, to ensure data anonymity.

### 3.3. Measures

The measure of each variable was translated from English into Amharic by a senior university lecturer. Independent English back-translations were subsequently performed by two English language lecturers who were fluent in English and native to Amharic. Although a few minor discrepancies were observed between the translators, they reached a consensus through discussion. Minor wording adjustments were made to enhance clarity and ensure that the items were easily understood by Ethiopian high school students, while still accurately reflecting local educational practices. Moreover, the instruments used have previously been employed in local studies with acceptable levels of reliability, further supporting their cultural appropriateness.

Confirmatory factor analysis (CFA) was run to examine the psychometric properties of the measures and ensure that each scale provides an accurate and reliable measure of the intended construct. Model fit was assessed using CFI/TLI (≥0.90 for good, ≥0.95 for excellent), RMSEA (<0.06 for excellent, < 0.08 for acceptable), SRMR (≤0.08 for good fit) (Hu & Bentler, 1999), and the chi-square ratio (χ^2^/df), where <3 indicates a good fit (Kline, 2011) and up to 5 is acceptable (Schumacker & Lomax, 2004).

#### 3.3.1. Academic Engagement

Skinner et al.’s (2009) engagement measure was used to assess the behavioral and emotional aspects of engagement. Two extra items from M.-T. Wang et al. (2016) were added to the behavioral subscale. While the behavioral engagement scale consists of six items (e.g., “In class, I work as hard as I can”), emotional engagement includes five items (e.g., “When I work on my schoolwork, I feel good”). The cognitive engagement dimension has five items (such as “Time flies when I’m studying”); three of them are the Schoolwork Engagement Inventory’s absorption subscale (Salmela-Aro & Upadaya, 2012), and the other two are from Ben-Eliyahu et al.’s (2018) cognitive engagement scale to be in line with the conceptualization of the variable and the recommendation forwarded by Wong and Liem (2022). The items were with a 5-point Likert-type scale, ranging from 1 (“not at all true for me”) to 5 (“very true for me”). In prior research (Salmela-Aro & Upadaya, 2012; Skinner et al., 2009), each dimension with its original items received adequate reliability estimates. For the present 16-item academic engagement measure, both Woreta (2024) and the present study reported good reliability evidence (α = 0.89–0.91). In the case of CFA, the current study revealed an excellent fit to the three-dimensional model of academic engagement (χ^2^/df, = 3.1, TLI = 0.95, CFI = 0.96, RMSEA = 0.06, and SRMR = 0.036).

#### 3.3.2. Peer Effort Socialization

To capture a good picture of the participants’ peer context, they were instructed (a) to list friends (students) with whom they spend more time and engage in various activities with; (b) the individuals they listed had to be in the same grade level, regardless of their schools or classrooms; and (c) the students in this group were called “your friends or friendship group” across all items used to measure peer context-related variables.

Peer effort socialization was assessed using a 4-item scale modified from the effort subscale of the Educational Socialization Scale (Bempechat et al., 1999), with response options ranging from 1 (not at all) to 5 (always). A sample item is, “My friends say we could do better in school if we worked harder”. The measure demonstrated good internal consistency both in the prior study (Woreta, 2024; α = 0.77) and in the present sample (α = 0.79). Fit indices based on CFA also collectively indicate an acceptable fit between the model and the data: χ^2^/df = 1.5, TLI = 0.98, CFI = 0.99, RMSEA = 0.03 (90% CI = 0.001, 0.092), and SRMR = 0.011.

#### 3.3.3. Peers’ Academic Norms

A 7-item scale derived from the Peers’ Academic Support and Aspirations Scale (Murdock, 1999; Murdock et al., 2000) was used to measure the academic norms of peer groups. The items ask participants, with a rating scale from 1 (not at all) to 5 (always), to what extent most of the members of their peer group had the potential to do a variety of academic tasks and demonstrate positive academic behaviors relevant to high school education. A sample item is “Most of my friends try to do well in school”. In a prior study (Woreta, 2024; α = 0.84), as well as in the present sample (α = 0.85), strong reliability evidence was obtained. Fit indices derived from CFA also collectively indicate an acceptable model-data fit: χ^2^/df = 2.9, TLI = 0.969, CFI = 0.979, RMSEA = 0.057 (90% CI = 0.037, 0.078), and SRMR = 0.029.

#### 3.3.4. Peers’ Educational Aspirations

A 5-item peers’ educational aspiration scale, adapted from the Peers’ Academic Support and Aspirations Scale (Murdock, 1999; Murdock et al., 2000), was used to assess students’ perceptions of their friends’ likelihood of graduating high school and pursuing further education. In other words, students responded to items evaluating the academic future of their peers. Sample item: “Most of my friends plan to go to college/university”. The rating scale ranges from 1 (not at all) to 5 (always). The scale demonstrates strong internal consistency (α = 0.82; Woreta, 2024) and in the present sample (α = 0.87). The CFA results provided fit indices that collectively indicate an acceptable fit between the model and the data: χ^2^/df = 2.7, TLI = 0.98, CFI = 0.99, RMSEA = 0.054 (90% CI = 0.02, 0.089), and SRMR = 0.016.

#### 3.3.5. Academic Self-Efficacy

The participants’ academic efficacy beliefs were assessed using the eight-item academic self-efficacy subscale of the self-efficacy questionnaire for children (SEQ-C; Muris, 2001), with a four-point Likert-type scale ranging from “not at all confident” (1) to very confident (4). A sample item is “How confident are you that you could study when there are other interesting things to do?” This measure has shown strong internal consistency evidence in prior studies (e.g., Landon et al., 2007; Minter & Pritzker, 2017; Suldo & Shaffer, 2007; Woreta, 2024), which is also excellent for the current participants (α = 0.926). CFA also indicated that the model fits the data (χ^2^/df = 3, SRMR = 0.02, RMSEA = 0.059, [90% CI = 0.043–0.076], CFI = 0.98, and TLI = 0.98).

#### 3.3.6. Educational Outcome Expectations

A 14-item scale, which was adapted by Woreta (2024) from the 19-item College Outcome Expectations Questionnaire (Flores et al., 2008), was used to assess the outcomes the students associate with pursuing secondary and postsecondary education. In adapting the items for this study’s student population, the stem “A college education will ...” was replaced by “Secondary and postsecondary education will …” Each item (e.g., “Secondary and postsecondary education will allow me to obtain a well-paying job”) was rated on a 5-point scale ranging from strongly disagree = 1 to strongly agree = 5; high values reflected higher positive educational outcome expectations. This 14-item scale has an excellent internal consistency, with Woreta (2024) reporting a 0.94 Cronbach’s alpha, while the present study found a value of 0.92. The CFA results supported the scale’s unidimensionality (χ^2^/df = 3, TLI = 0.96, CFI = 0.97, RMSEA = 0.059 [90% CI = 0.050–0.067], and SRMR = 0.028).

#### 3.3.7. Covariates

As prior evidence suggests, the effects of gender and socioeconomic status (SES) on academic engagement and self-efficacy (e.g., Else-Quest et al., 2010; Li et al., 2011; Skinner et al., 2009; M.-T. Wang et al., 2019a, 2019b; Yu et al., 2019) were included as covariates in the current model to account for their potential influence. Participants indicated the highest level of education that their mother and father had attained (1 = illiterate, 2 = primary school, 3 = middle school, 4 = high school, 5 = diploma, 6 = undergraduate degree, 7 = master’s and above). They also completed a four-item Family Affluence Scale (FAS II: Currie et al., 1997) which includes items such as “Does your family own a car, van or truck?”. The response options were 0 = none (no), 1 = once (one), and 2 = more than once (one), but one item was recorded as yes (=1) or no (=0). Hence, SES was assessed by combining parents’ level of education and the Family Affluence Scale. Concerning gender, female and male are represented by 1 and 2, respectively.

## 4. Results

### 4.1. Preliminary Analyses

The present data met both the outliers and normality assumptions because: (a) the numerical normal deviates method provided the absolute value of a z-score less than 3.0, which means there were no univariate outliers (Kline, 2023); (b) there was no evidence of multivariate outliers (i.e., a small value of the Mahalonbis distance *D*^2^ and the lowest *p*-value, 0.001); (c) the highest absolute value of skewness was 0.43, and for kurtosis, it was 0.62, which means univariate normality is not an issue (Finney & DiStefano, 2013; Kline, 2023); and (d) multivariate kurtosis (Marida’s normalized estimate for multivariate normality) was three, which is less than five (Byrne, 2016). Moreover, the variables’ correlations ranged from 0.26 to 0.62, indicating that multicollinearity was not a concern in the present datasets (Kline, 2023). Moreover, to ensure that the independent variables were adequately distinct from each other, multicollinearity diagnostics were performed. The tolerance values for all predictors ranged from 0.470 to 0.790, surpassing the recommended minimum of 0.10. Additionally, the variance inflation factor (VIF) values ranged from 1.265 to 2.127, which are significantly below the commonly accepted maximum of 5. Therefore, multicollinearity is not an issue, allowing each predictor to provide unique variance in explaining the outcome variable (Hair et al., 2019; Kline, 2023). Hence, no cases or variables were deleted in the process of preliminary analysis. Table 1 displays the descriptive statistics and correlations of the study variables.

Regarding common methods bias, in addition to procedural efforts aimed at mitigating potential biases, such as ensuring participant anonymity, Harman’s one-factor test was conducted. The results indicated that the first factor accounted for 27.120% of the total variance, which is substantially below the threshold of 50%. This suggests that common method bias is unlikely to pose a threat to the validity of the findings.

### 4.2. Primary Analysis

**Figure 2:**
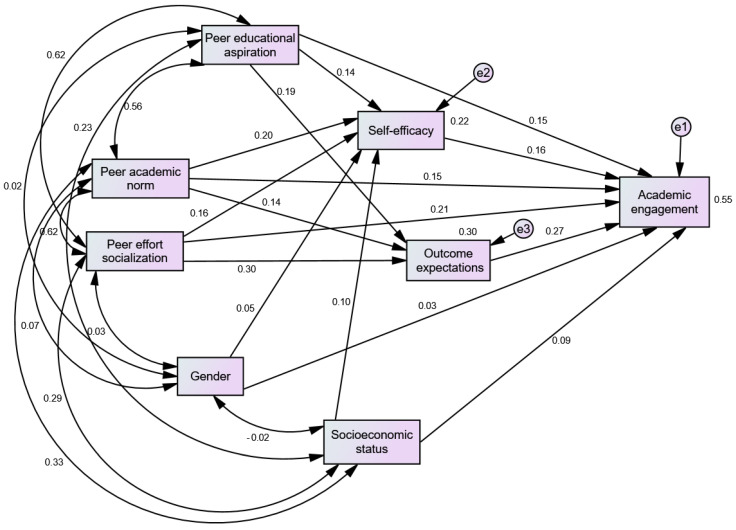
Peer matter engagement model.

As the data met the method’s basic assumptions, SEM with the maximum likelihood estimation method was used to estimate the parameters of the model, using IBM Amos (Version 23) statistical software. The results of the analysis have shown an excellent model fit to the data (χ^2^ = 4.24, df = 3, *p* = 0.237, TLI = 99, CFI = 0.99, RMSEA = 0.026 [90% CI = 0.000–0.078], and SRMS = 0.012). The paths posited within the structural model were statistically significant and the directions of the relations are as predicted, except for those linking gender (covariate) to the mediator and outcome variables. The standardized direct effects and the variances accounted for are shown in Figure 2. Paths linking peer academic norms to students’ academic engagement (β = 0.15, *p* < 0.001), outcome expectations (β = 0.14, *p* < 0.01), and academic self-efficacy (β = 0.20, *p* < 0.001) were positive and significant. Peer effort socialization positively predicted students’ academic self-efficacy (β = 0.16, *p* < 0.01), outcome expectations (β = 0.30, *p* < 0.001), and academic engagement (β = 0.21, *p* < 0.001). Peer educational aspiration positively influenced students’ self-efficacy (β = 0.14, *p* < 0.01), outcome expectations (β = 0.19, *p* < 0.001), and academic engagement (β = 0.15, *p* < 0.001). Together, peer context variables explained a significant amount of the variance in academic self-efficacy (22%) and educational outcome expectations (30%). Both self-efficacy (β = 0.16, *p* < 0.001) and outcome expectations (β = 0.27, *p* < 0.001) were also related to academic engagement. The model’s variables collectively explained 55% of the variation in high school students’ academic engagement. The results also indicate that peer effort socialization had the strongest positive effects on students’ academic engagement (β = 0.21, *p* < 0.001) and outcome expectations (β = 0.30, *p* < 0.001). In terms of shaping students’ beliefs in their academic abilities, peers’ academic norms were found to contribute more (β = 0.20, *p* < 0.001).

### 4.3. Mediated Effects

To examine the indirect effects of peers’ academic norms, educational aspirations, and efforts on academic engagement (via outcome expectations and self-efficacy), bootstrap analysis with 5000 bootstrap samples (Collier, 2020; Fairchild & McQuillin, 2010) was conducted. As presented in Table 2, the results of the bootstrap analysis revealed that academic self-efficacy (b = 0.022, BC 95% CI = 0.008, 0.041, *p* < 0.01) and outcome expectations (b = 0.053, BC 95% CI [0.028, 0.086], *p* < 0.001) partially mediated the relationship between peer educational aspirations and high school students’ academic engagement. The partial mediated effects of peers’ academic norms on high school students’ academic engagement via self-efficacy (b = 0.030, BC 95% CI= 0.014, 0.054, *p* <.001) and outcome expectations (b = 0.037, BC 95% CI [0.014, 0.062], *p* < 0.01) were significant. Mediated by academic self-efficacy (b = 0.024, BC 95% CI = 0.009, 0.044, *p* < 0.01) and outcome expectations (b = 0.078, BC 95% CI [0.050, 0.112], *p* < 0.001), peer effort socialization was found to have significant and positive indirect effects on high school students’ academic engagement. Overall, the mediation analysis revealed that outcome expectations and self-efficacy partially mediated the link between high school students’ academic engagement and their peer context.

While several mediation paths reached statistical significance (Table 2), the practical significance of these effects varies. For example, the indirect effect of positive emotional appraisal (PEA) on academic achievement through self-efficacy was β = 0.022, and through outcome expectations was β = 0.053, both statistically significant but considered small in size. Similarly, the indirect effects of positive academic norms (PAN) and positive emotional support (PES) through these mediators ranged from β = 0.024 to β = 0.078, also falling into the small effect size category. According to Cohen’s (1988) benchmarks, effects below 0.10 are considered small and may be imperceptible in daily classroom contexts. Therefore, while our findings provide evidence of mediation, the magnitude of these effects suggests that interventions should target the larger pathways, such as the combined sequential mediations (e.g., PES → SE → OE → AE, β = 0.101), though still modest, to approach a more meaningful level of practical significance in educational settings.

## 5. Discussion

The purpose of this study was to examine how peer context variables (academic norms, educational aspirations, and effort socialization), self-efficacy, and outcome expectations shape high school students’ academic engagement. The results revealed that the proposed model fit the data well, all paths of interest were significant, the model’s variables explained 55% of the variance in academic engagement, and the Social Cognitive Theory-based hypotheses were supported. While peer effort socialization had the strongest positive effects on students’ academic engagement and outcome expectations, peers’ academic norms were found to contribute more to shaping students’ beliefs in their academic abilities.

In this study, the academic norms, educational aspirations, and efforts prevailing in peer groups directly influenced high school students’ academic engagement. This finding supports the idea that peers play an important role in the development of adolescents (Furrer, 2010; Wentzel, 2009), and that the peers can have direct influences through modeling, reinforcement, encouragement, or pressures to adhere to group norms (Altermatt & Pomerantz, 2003; Lynch et al., 2013).

The findings of the present study also substantiate prior studies that found peers are alike in various ways, such as motivation to learn (Kindermann et al., 1996; Ryan, 2001), putting in efforts (working hard), and having an intrinsic value, interest, and enjoyment in school tasks (Ryan, 2001; Shin & Ryan, 2014). A recent study also found that peer academic socialization has a direct influence on academic engagement (Woreta, 2024). So, this study’s findings corroborate previous studies and enhance the theoretical assumptions that peers play a tremendous role in socializing each other’s behaviors and that others matter in cultivating student engagement and motivation in education. An encouraging finding of the present study is that a peer context characterized by high educational aspirations, positive academic norms, and an emphasis on effort plays a significant role in shaping students’ academic engagement, supporting the broader theoretical understanding that peer socialization is a critical factor in promoting academic development. Peers who value academic success and model positive academic behaviors contribute to fostering higher levels of engagement among students. Stating differently, when students are surrounded by peers who prioritize academic achievement and exhibit constructive academic behaviors, they are more likely to adopt similar attitudes and practices.

### Self-Efficacy and Outcome Expectations as Mediators

This study’s findings support the hypotheses that both self-efficacy and outcome expectations mediate the relationships between the peer context and learning engagement. According to the results, high school students in a positive peer context—where academic norms are encouraging, peers have high educational aspirations, and academic effort is emphasized—demonstrated higher academic efficacy beliefs and expected positive outcomes from their education. In turn, these motivational beliefs led to a greater degree of engagement in academic activities. The Social Cognitive Theory’s hypothesis that if students have strong beliefs in their academic capabilities and anticipate positive outcomes from their education, they will show greater engagement in their learning was supported (Bandura, 1986, 1997; Lent et al., 1994). The finding that academic self-efficacy influenced students’ engagement was consistent with prior empirical evidence (Linnenbrink & Pintrich, 2003; R. D. Liu et al., 2018; Martin & Rimm-Kaufman, 2015; Navarro et al., 2019; Rimm-Kaufman et al., 2015). As students with strong academic self-efficacy exhibit greater confidence in completing learning tasks, they demonstrate higher levels of academic engagement (Akbari et al., 2025; Guo et al., 2025; Huang & Wang, 2023; Lin, 2021; Q. Liu et al., 2023; Meng & Zhang, 2023; Shao et al., 2025; Y. Wang & Zhang, 2024), whereas students with lower academic self-efficacy are more likely to experience feelings of helplessness, heightened negative emotions, and diminished academic engagement. The positive influence of outcome expectations on students’ academic engagement observed in the present sample is consistent with the prior findings (Miller et al., 2021; Navarro et al., 2019; Woreta, 2024). Prior studies grounded in expectancy–value theory have also demonstrated that the utility value directly predicts students’ engagement in learning (Goto, 2024; Hidayatullah et al., 2023; Sasidharan & Kareem, 2023), highlighting that when students perceive the relevance and usefulness of learning, their engagement levels increase. Similarly, the perceived value of effort, conceptualized as an outcome expectation reflecting students’ subjective perceptions of the usefulness of trying hard in school, has also been found to predict academic engagement (Xie & He, 2023).

The results of this study indicate that students in a peer group with more positive and motivating academic norms, educational aspirations, and effortful behaviors have strong beliefs in their abilities to perform well in schools and believe that pursuing education will lead to the outcomes they value. The finding that peers’ academic norms, educational aspirations, and effort socialization all predict educational outcome expectations suggests that, during adolescence, peers provide an actual environment for envisioning and planning the future and are a crucial source of future-focused insights (Dawkins et al., 2021; Malmberg, 1996). Another key insight from this study is that similar learning experiences can shape both self-efficacy and outcome expectations, which in turn influence outcome variables in a comparable manner (Bandura, 1997; Lent et al., 1994; M.-J. Liu et al., 2022).

Self-efficacy and outcome expectations partially mediated the relationship between peer-related variables and students’ academic engagement, supporting the social cognitive perspective that the social context operates through personal factors, such as self-efficacy beliefs and outcome expectations to produce the required outcomes, such as academic engagement (Bandura, 1997; Lent et al., 1994). According to this finding, the peer context enhances academic engagement by influencing self-efficacy and outcome expectations through its various dimensions. That is, students who perceived a positive academic peer context had higher beliefs in their abilities (self-efficacy), associated positive outcome expectations with their education, and demonstrated better learning engagement. These findings are in line with prior studies in Malaysia (Benlahcene et al., 2024) and China (Shao & Kang, 2022), where self-efficacy and outcome expectations were found to mediate the link between peer factors and academic engagement. However, unlike the Chinese context where academic resilience also played a central role, our findings in Ethiopia highlight the primacy of peer effort socialization and outcome expectations, possibly due to the higher reliance on peer networks in under-resourced settings. This highlights the cultural and systematic variations that shape the functioning of SET constructs across different societies.

The prior literature documented that people in the same peer group: (a) are alike in expectations about their future education (Kiuru et al., 2007), (b) are essential sources of information about the future (Malmberg, 1996), (c) are role models for each other (Bandura, 1986, 1997), (d) want to adhere and fit the group’s norms (M.-T. Wang & Eccles, 2012), and (e) tend to adjust their engagement levels to match those of their peers (M.-T. Wang et al., 2018). All of these could explain how peer context factors influence student learning engagement through multiple pathways and how the effects might be channeled. This study’s findings, along with past evidence, suggest that the peer context is a vital substrate for enhancing students’ academic engagement, particularly in a collectivistic culture like Ethiopia. Overall, social cognitive theory, which posits that an individual’s behaviors are shaped by social environmental and personal factors (Schunk & DiBenedetto, 2020), is supported by the present study.

It may be worthy enough to note that understanding the role of the peer context in shaping individuals’ beliefs, attitudes, and behaviors requires the careful consideration of the cultural context, as cultural values fundamentally shape peer relationships and the mechanisms through which the peer influence operates (Chen et al., 2006). In collectivistic cultures, such as Ethiopia, where interdependence, belongingness, group cohesion, cooperation, and social harmony are highly valued, peer relationships are deeply embedded in the communal fabric (Triandis, 1995). These relationships emphasize mutual support, shared responsibilities, and conformity to group norms, creating a socially cohesive environment that fosters academic encouragement and shared aspirations. Peers often function as role models, with their behaviors, study habits, and attitudes serving as benchmarks for others. A recent Ethiopian study, for example, found that peer academic socialization significantly predicts students’ academic engagement, underscoring the powerful role of peers in shaping educational outcomes (Woreta, 2024). In such contexts, reciprocity and mutual directionality further reinforce behavioral change, as students internalize collective expectations and align themselves with group-defined standards (Markus & Kitayama, 1991). Peer influence tends to be more pronounced in collectivistic cultures than in individualistic ones due to the stronger emphasis on social conformity and connectedness. Ethiopian culture is collectivistic, where connectedness, interdependence, and conformity are given more attention; consequently, students can be strongly influenced by their perceptions of peer expectations, often aligning their beliefs, attitudes, and behaviors with those of their peer group to maintain group cohesion and social approval.

It is also important to consider the broader Ethiopian socio-political and educational context to contextualize the findings, as this would be helpful for practical actions beyond an enriching scholarship in the area. According to Bandura’s social cognitive theory (1997), the social environment plays a key role in influencing how people act and think, with the larger political and educational context affecting how students view their relationships with peers, assess their confidence in their abilities, understand what to expect from their academic efforts, and interact with their education.

In Ethiopia, the political environment has historically shaped the goals, governance structures, and implementation of education policy, which in turn affects students’ academic and psychosocial experiences (Gershberg et al., 2023). The country’s educational system has undergone significant shifts across regimes—from the Imperial era to the Derg and, more recently, the EPRDF and Prosperity Party administrations—resulting in weak policy continuity, with frequent overhauls linked to broader political transitions (Gershberg et al., 2023). Throughout these shifts, a priority on access has led to large class sizes, under-resourced schools, and higher student–teacher ratios (Ministry of Education, 2021). More recently, teacher demotivation and a declining professional status, exacerbated by inflation and weak career incentives, have created classroom conditions where affective and motivational support for students is often lacking. Consequently, students frequently depend on their peers for academic and emotional support, rendering the peer context a vital influence on the development of self-efficacy and outcome expectations, and ultimately, on academic engagement.

Another defining feature of the Ethiopian education system is the high-stakes nature of national examinations, which continue to function as gatekeepers for educational and occupational opportunities. This can affect the peer context and psychological processes, influencing students’ motivation and engagement. Moreover, pervasive concerns about future employability in the context of high youth unemployment further influence students’ motivation. In such settings, peer modeling and reinforcement become vital for meeting educational demands and planning for the future. Additionally, political instability and regional differences in resource allocation may moderate the effects observed in this study (Tareke et al., 2024). For example, students in conflict-affected or under-resourced areas may experience lower self-efficacy and engagement due to heightened stress and limited support, potentially weakening the pathways identified here.

## 6. Limitations and Future Research

This study has many remarkable strong points. For example, it addresses how each aspect of the peer context (i.e., peers’ academic norms, educational aspirations, and emphasis on effort) affects students’ academic engagement, which previous studies of peer influence have missed, including the way they have been measured in the present study. Furthermore, this study used randomly selected participants from different schools and was conducted in a setting where the issue had not received attention in the past. Important covariates, such as gender and the socioeconomic status, were also included. Nonetheless, like any other study, the following limitations are associated with this study.

The first limitation is that this study did not control for the influence of older siblings. For example, research has demonstrated that older siblings influence younger siblings’ academic performance and engagement (Bouchey et al., 2010; M.-T. Wang et al., 2019a, 2019b), which is also likely to influence students’ beliefs in their learning efficacy and in the expected outcomes. As a result, future researchers might be advised to consider the influence of older siblings and other social influences (e.g., teachers) on academic engagement and its immediate predictors, as this study has shown. Second, there is no doubt that (a) the model’s structural relations were based on well-known theory and previous empirical evidence, (b) SEM can suggest causal relationships, and (c) the findings converge with what the theory predicts and what previous studies have reported. Although all these conditions are true, this cross-sectional study still cannot establish causal ordering among the model’s variables. Hence, we found it logical to suggest longitudinal designs for future researchers to address such an important limitation. Third, motivational beliefs such as self-efficacy and outcome expectations can be best captured with self-report measures. However, for academic engagement and peer-related academic contexts, self-report measures may lead to social desirability biases. As a result, future researchers are encouraged to use either other alternative mechanisms or multiple sources of data to address such a key concern. Fourth, the other limitation of this study may be associated with the representativeness of the participants across the nation. While the participants are representative of students within the city or relative to the Amhara region, they may not accurately reflect the demographic diversity of students across the nation as a whole. So, while students in the country are more alike in many ways, the results may not fully represent and apply to other parts of Ethiopia or the national student population. Further research should aim to include a more diverse sample and a broader range of demographic variables to enhance the generalizability of the findings. Fifth, while the present study used bootstrapped path analysis to test mediation effects, we did not conduct a formal model comparison to test competing mediation structures (e.g., parallel vs sequential mediation models). Future studies should apply SEM or Bayesian methods to compare alternative models and assess the strength and fit of different mediation pathways. Sixth, the current study’s findings, therefore, should be interpreted in light of Ethiopia’s socio-political and educational realities. The political–educational context functions not simply as background, but as a structural moderator that shapes the dynamic relationships among peer interactions, motivational beliefs, and academic engagement. Thus, understanding the political–educational complexity is crucial for (a) grasping the nuances of a student engagement scholarship and (b) designing interventions that leverage peer dynamics to enhance motivational beliefs and foster engagement among students. Future research should explicitly examine these contextual factors or potential moderators.

Finally, it is essential to note that in psychological research, what occurs in one context may not occur in another (Linnenbrink & Pintrich, 2003). This suggests the need to examine this study’s model in different contexts to see how the results vary across different cultures and populations.

## 7. Conclusions and Implications

This study’s findings offer significant insight into how peer contexts shape students’ academic engagement by identifying the underlying mechanisms through which each aspect of it exerts influence, contributing to both theory and practice. This could help us learn more about peer-focused predictors and how they relate to academic engagement and forward the imperative of considering peer-related factors in learning environments, especially in the scholarship of adolescent student engagement. Scholars often advise testing the applicability of a given theory or model across cultures. This study suggests the applicability of social cognitive theory, originally developed in individualistic Western cultures, to collectivistic Ethiopian cultures. Based on the results, interventions that focus on peer contexts can be beneficial for enhancing students’ beliefs in their academic abilities and the positive outcomes of education, as well as for promoting learning engagement, beyond this study’s contribution to the advancement of theoretical knowledge. Together with this study’s results, the fact that self-efficacy and outcome expectations are malleable suggests that interventions that aim to enhance these personal factors are very helpful to engage students in their learning. The key insight here is that parents, teachers, and counselors can enhance students’ academic engagement by creating contexts that cultivate efficacy and outcome expectancy beliefs. According to this study’s findings, academic norms, educational aspirations, and conviction in the value of hard work prevailing in a particular group are core peer context variables that affect students’ learning engagement in a high school context. Interventions focusing on peer groups are likely to be more effective in enhancing high school students’ academic self-efficacy, educational outcome expectations, and engagement in learning, as adolescents are highly susceptible to peer groups’ beliefs and behaviors, including their academic performance and self-perceptions.

In a more specific saying, this study’s findings suggest that the positive academic behaviors exhibited by peers (peers’ academic norms) and the associating achievement with hard work (effort socialization) contribute more significantly to the levels of student engagement. This implies that interventions that prioritize fostering positive academic norms among peers, together with an emphasis on effort, could substantially enhance student engagement and improve learning outcomes. Therefore, educators and school counselors need to focus on fostering positive academic norms among peers through various strategies, such as promoting collaborative learning environments, acknowledging academic excellence, and modeling positive academic behaviors. Also, implementing growth mindset interventions, which inspire taking challenges as opportunities for development, and establishing consistent systems of recognition for persistence and effort-based success may lead to an enhanced emphasis on effort as these can underscore the value of hard work and resilience in the learning process.

The findings of this study, although conducted in the collectivistic culture of Ethiopia, align with the predictions posited in SCT. Also, this study gives us new insights into how and to what extent each aspect of the peer context influences students’ academic engagement, both directly and indirectly via person–cognitive variables. Furthermore, this study highlights that the relationships between peer context variables (i.e., academic norms, educational aspirations, and effort socialization) and academic engagement were partially mediated by self-efficacy and outcome expectations, underscoring the need to consider these factors when individuals (e.g., parents, educators, and psychologists) design and implement interventions aimed at enhancing students’ active involvement in their academic tasks.

Given the collectivistic culture of this study’s population, group-focused interventions would help boost students’ motivational beliefs (e.g., self-efficacy and outcome expectations) and academic engagement in learning. The evidence has shown that cooperative learning can enhance positive peer relationships (Roseth et al., 2008; Van Ryzin & Roseth, 2019), which would be particularly important in collectivist cultures like Ethiopia. The perspective that schools are important contexts for adolescent development (Eccles & Roeser, 2011) suggests that cultivating peer groups that pay due attention to academic affairs would be helpful, and such an endeavor actually requires intentional efforts to connect students with peers who actively engage in their learning and aspire to pursue a post-high school education. This approach creates opportunities for students to observe and interact with motivated peers, thus inspiring them to have greater educational aspirations and expectations through social exposure and modeling (Schunk & DiBenedetto, 2020; Usher & Pajares, 2008). Educators can strengthen collaborative learning by integrating group-based projects and activities. When students observe peers engaging persistently with their academic tasks, they are more likely to build confidence in their abilities and anticipate positive outcome expectations, thereby increasing their level of engagement. Peer mentorship programs, which facilitate knowledge sharing, encouragement, and exposure to diverse peers, can help schools cultivate students’ academic motivation and engagement. These programs are essential in shaping students’ beliefs, attitudes, and behaviors in educational settings. By embedding peer collaboration and mentorship into the school policy and everyday practice, educational systems can create socially enriched learning environments that support both academic engagement and achievement. The aforementioned mechanisms can create rich environments for peer socialization in academic contexts.

## Figures and Tables

**Table 1:** Descriptive statistics and correlations of variables.

	Variables	1	2	3	4	5	6
1	Peers’ academic norms	_					
2	Peers’ educational aspirations	0.56 **	_				
3	Academic engagement	0.57 **	0.56 **	_			
4	Peer effort socialization	0.62 **	0.61 **	0.62 **	_		
5	Academic self-efficacy	0.41 **	0.37 **	0.45 **	0.39 **	_	
6	Outcome expectations	0.44 **	0.46 **	0.57 **	0.51 **	0.26 **	_
	Mean	3.34	3.52	3.46	3.42	2.43	3.69
	Standard deviation	0.77	0.74	0.75	0.79	0.58	0.66
	Shewness	−0.22	−0.43	−0.04	−0.13	0.12	−0.42
	Kurtosis	−0.40	−0.25	−0.62	−0.56	−0.37	−0.40

Note: N = 596, ** *p* < 0.01 (2-tailed).

**Table 2:** Direct, indirect, and total effects.

Independent Variable	Direct Effects				Indirect Effects				Total Effect
	Mediator/s	Dependent Variable	Estimate	*p* Value	Estimate	*p* Value	Bias-Corrected 95% CI		Estimate
							Low	High	
PEA →	-	SE	0.107 (0.14)	0.005	-	-	-	-	0.107 (0.14)
PAN →	-	SE	0.150 (0.20)	0.000	-	-	-	-	0.150 (0.20)
PES →	-	SE	0.117 (0.16)	0.002			-	-	0.117 (0.16)
PEA →	-	OE	0.173 (0.194)	0.000			-	-	0.173 (0.194)
PAN →	-	OE	0.119 (0.14)	0.002			-	-	0.119 (0.14)
PES →	-	OE	0.252 (0.30)	0.000			-	-	0.252 (0.30)
SE →	-	AE	0.201 (0.16)	0.000	-	-			0.201 (0.16)
OE →	-	AE	0.309 (0.27)	0.000					0.309 (0.27)
PEA →	SE →	AE	0.152 (0.15)	0.000	0.022 (0.022)	0.002	0.008	0.041	0.174 (0.172)
PAN →	SE →	AE	0.145 (0.15)	0.000	0.03 (0.032)	0.000	0.014	0.052	0.175 (0.182)
PES →	SE →	AE	0.198 (0.21)	0.000	0.024 (0.026)	0.002	0.009	0.045	0.222 (0.236)
PEA →	OE →	AE	0.152 (0.15)	0.000	0.053 (0.051)	0.000	0.028	0.086	0.205 (0.201)
PAN →	OE →	AE	0.145 (0.15)	0.000	0.037 (0.039)	0.002	0.014	0.063	0.182 (0.189)
PES →	OE →	AE	0.198 (0.21)	0.000	0.078 (0.081)	0.000	0.050	0.113	0.276 (0.291)
SE →	-	AE	0.201 (0.15)	0.000	-				0.201 (0.15)
OE →	-	AE	0.309 (0.27)	0.000	-				0.309 (0.27)
PEA →	SE →OE →	AE	0.152 (0.15)	0.000	0.075 (0.074)				0.227 (0.222)
PAN →	SE →OE →	AE	0.145 (0.15)	0.000	0.067 (0.069)				0.212 (0.217)
PES →	SE →OE →	AE	0.198 (0.21)	0.000	0.101 (0.106)				0.300 (0.313)

Note. PAN = peers’ academic norms; PEA = peers’ educational aspirations; PES = peer effort socialization; AE = academic engagement; SE = self-efficacy; OE = Outcome expectations. Values in parenthesis are standardized coefficients.

## Data Availability

The corresponding authors hold the datasets generated and analyzed during this study and are willing to share them upon request.
